# Algorithm for empirical glycopeptide treatment in patients with hematologic malignancies and enterococcus faecium blood stream infection

**DOI:** 10.1186/1753-6561-5-S6-P49

**Published:** 2011-06-29

**Authors:** X Zhou, M Kerkhof, L Span, A Friedrich, J Arends

**Affiliations:** 1 Medical microbiology, University Medical Centre Groningen, Groningen, Netherlands; 2 Epidemiology, University Medical Centre Groningen, Groningen, Netherlands; 3 Hematology, University Medical Centre Groningen, Groningen, Netherlands

## Introduction / objectives

*Enterococcus faecium* has become a major cause of nosocomial infections especially in patients with hematologic malignancies. The aim of this study was to determine risk factors in those patients who are at risk of *Enterococcus faecium* blood stream infection (BSI) and should be considered for empirical treatment.

## Methods

Retrospectively demographic, clinical and microbiological data in 33 patients with an *E. faecium* BSI were compared to 66 control patients during a 5-year period at the hematology ward. Multivariate logistic regression was used to explore the independent risk factors in order to develop a prognostic model to determine the risk of *E. faecium* BSI.

## Results

Significant associations of *E.faecium* BSI were found with age, hospital stay prior to blood culture, duration of hospitalization 1 year before admission, fever prior to blood culture, severity and duration of neutropenia, CRP (C-reactive protein) at time of blood culture withdrawal, colonization with *E. faecium* prior to blood culture and diarrhea. *E. faecium* BSIs were found associated with more severe disease and higher mortality rates. Independent risk factors for *E. faecium* BSI were colonization with *E. faecium* 30 days prior to blood culture (OR 3.83; CI 1.1-12.8), fever > 1 day (4.02; 1.3-12.8), hospital stay prior to blood culture > 14 days (4.78; 1.3 -18.0), age > 59 years (5.47; 1.6-18.2) and abdominal pain, diarrhea or neutropenia (5.95; 1.1-31.4).

## Conclusion

Using prognostic modeling, risk stratification is possible for development of *E. faecium* BSI in patients with hematological malignancies. Empirical treatment should be considered in patients who are at high risk.

## Disclosure of interest

None declared.

